# Notes and News

**Published:** 1895-04-13

**Authors:** 


### Notes and News.

Collected and Communicated.

Lord Iveagh has given a donation of £1,000 to the St. Thomas's Special Appeal Fund.

It is announced that Dr. Patrick Manson, LL.D., M.D., has been elected to the post of Lecturer on Tropical Diseases at St. George's Hospital. The appointment is interesting as being the first provision made in London for special instruction in tropical medicine, the first attempt to fill the void in medical education to which we drew attention in The Hospital of January 26.

Sir J. Russell Reynolds, M.D., F.R.S., has been re-elected President of the Royal College of Physicians.

It is satisfactory to find from recent statistics that the town of Worthing has more than regained its prestige as one of the healthiest of seaside resorts, so rudely broken by the terrible epidemic of two years since. The death rate during 1894 is stated to have been 12·5, whilst in the case of the 33 other large towns of which the Registrar-General's returns have been so far published, the rates have ranged from 13·1 to 23·8. Under these circumstances it is to be hoped that the forthcoming season may do something to compensate the people of Worthing for the trouble and loss of which they have had so large a share of late.

The memory of the late Professor Swing is to be perpetuated in Chicago by the building of an Emergency Hospital, a scheme which this physician himself had much at heart. The erection of an Infectious Hospital in the neighbourhood of Baltimore is again under consideration, the plan proposed last year having remained unfulfilled in consequence of the opposition of many residents. Another new hospital is the one with which Mr. and Mrs. William D. Sloane have generously undertaken to replace the old buildings of the Maternity Hospital, N.Y.

At a meeting of the Council of the Royal College of Surgeons of England, on Thursday, April 4th, Mr. Christopher Heath was elected President of the College, in succession to the late Mr. J. W. Hulke, for the remainder of the collegiate year, that is until July 11th next.

We understand that the Judges of the Chancery Division have decided to pay the interest on a sum of upwards of £40,000 in their hands to the Metropolitan Sunday Fund for distribution amongst the hospitals every year. We also understand that a gentleman, whose name cannot be mentioned at present, is desirous of giving £200,000 to be invested, in the names of trustees, the interest of which is to be paid yearly to the Council of the Metropolitan Sunday Fund, who are to distribute it amongst the hospitals at their discretion. We congratulate the Council upon these signal marks of public confidence, and would especially tender the thanks of all hospital managers to Mr. Henry N. Custance, their secretary, who has shown marked ability and discretion throughout the preliminary negotiations.

A new plan for helping the funds of the Women's and Children's Hospital was evolved the other day by the energetic owner of *The Syracuse Post*. He lent "the plant" for twenty-four hours to a party of ladies, with permission to print as many editions and as large a paper as they could get through in the stated time, on the sole condition that everything except running the machinery was to be done entirely by women. The first copy printed was sold for fifty cents, the others for five cents. Much interest was taken in the enterprise, and of the ten thousand dollars cleared, five thousand were made by the insertion of advertisements. Syracuse is a city of only 100,000 inhabitants, and the sale of this paper was carried on in the streets by society ladies and young girls. The quantity of manuscript sent in was overwhelming, and as hardly one of the contributors had any previous knowledge of journalism, the amateur editors must have had an unenviable task.

We have received by mail a copy of *The Madagascar News* for February 16th and 23rd, containing an account of the spirited speech made to her people by H.M. Queen Rananalomanjaka, which awoke the most intense enthusiasm amongst the loyal crowds gathered for the occasion in Antananarivo. The scene must have been one never to be forgotten by those who witnessed it, and we hope that some measure of sympathy may be aroused in England to respond to an appeal for supplies of nursing appliances which is being made by Mr. Bennet Burleigh, who is acting as war correspondent in Madagascar. He states that when the war begins thousands of natives must perish for want of such simple requisites as quinine and surgical dressings. We have no doubt that these facts have merely to be stated to secure many subscriptions towards a fund for this purpose, which will be gratefully received and acknowledged by Miss Constance Williams, 1a, Dennington Park Mansions, West Hampstead, N.W., who is interesting herself in the matter as Mr. Bennet Burleigh's representative.

